# Prostaglandin E1 Attenuates Pulmonary Artery Remodeling by Activating Phosphorylation of CREB and the PTEN Signaling Pathway

**DOI:** 10.1038/s41598-017-09707-y

**Published:** 2017-08-30

**Authors:** Ying-Ju Lai, Hsao-Hsun Hsu, Gwo-Jyh Chang, Shu-Hui Lin, Wei-Jan Chen, Chung-Chi Huang, Jong-Hwei S. Pang

**Affiliations:** 1 Department of Respiratory Therapy, Chang Gung University College of Medicine, Tao-Yuan, 33353 Taiwan; 2 Cardiovascular Division, Chang Gung Memorial Hospital, Tao-Yuan, 33353 Taiwan; 3 Division of Thoracic Surgery, Department of Surgery, National Taiwan University Hospital and National Taiwan University College of Medicine, Taipei, 10002 Taiwan; 4 Graduate Institute of Clinical Medical Sciences, Chang Gung University College of Medicine, Tao-Yuan, 33353 Taiwan; 5 Division of Thoracic Medicine, Chang Gung Memorial Hospital, Tao-Yuan, 33353 Taiwan; 6 Respiratory Care, Chang-Gung University of Science and Technology, Chia-Yi, 61363 Taiwan; 7 Department of Physical Medicine and Rehabilitation, Chang Gung Memorial Hospital, Linkou Taoyuan City, Taiwan

## Abstract

The depletion of cyclic adenosine monophosphate (cAMP) response element binding protein (CREB) and phosphatase and tensin homolog (PTEN) is the critical mediator of pulmonary arterial hypertension (PAH). We hypothesized that the activation of phosphorylated CREB (pCREB) and PTEN could inhibit the AKT signaling pathway to attenuate pulmonary arterial remodeling in rats with monocrotaline-induced PAH. We observed decreased PTEN and pCREB in idiopathic PAH versus control tissue. We reduced PTEN using small interfering RNA in human control pulmonary arterial smooth muscle cells (PASMCs) and observed an increase in pAKT. Consistent with PTEN knockdown in PASMCs, prostaglandin E1 (PGE1) induced pCREB expression to stimulate PTEN protein expression and inhibited pAKT in a time- and dose-dependent manner. The enhanced proliferation and migration of PASMCs following PTEN knockdown were significantly inhibited by PGE1 treatment. The PGE1-induced elevation of PTEN expression in PTEN-depleted PASMCs was decreased by the application of a PKA inhibitor and a CBP-CREB interaction inhibitor. Supplementation with a novel emulsion composition comprising PGE1 in rats with monocrotaline-induced PAH prevented pulmonary arterial remodeling and improved hemodynamics via the induced expression of PTEN. We conclude that PGE1 recruits pCREB/PTEN to decrease the migration and proliferation of PASMCs associated with PAH. This finding elucidates a relevant underlying mechanism of the PGE1/CREB/PTEN signaling pathway to prevent progressive PAH.

## Introduction

Clinically defined pulmonary hypertension (PH) requires an increase in pulmonary artery pressure of more than 25 mm Hg that lead to right heart failure^1^. The hallmark of vascular remodeling in PH involves the migration and proliferation of vascular cells, particularly pulmonary arterial smooth muscle cells (PASMCs), which contribute to abnormal extracellular matrix assembly and muscularization of small pulmonary arteries^2,3^. Recent studies have reported that multiple factors drive PH, including bone morphogenetic protein receptor type II (BMPRII)^4,5^, platelet-derived growth factor (PDGF)^6^, neurogenic locus notch homolog protein 3 (NOTCH3)^7^ and forkhead box protein O1 (FoxO1)^8^, and an imbalance in prostacyclin signaling^9,10^.

Prostaglandins (prostaglandin I 2 [PGI2] and prostaglandin E 1 [PGE1]) are naturally occurring prostanoids that are endogenously produced as metabolites of arachidonic acid in the vascular endothelium^11^. In vascular smooth muscle cells, prostaglandin stimulates adenylate cyclase which converts adenosine triphosphate to cyclic adenosine monophosphate (cAMP) to increase intracellular cAMP levels^12^. Thus, the protein kinase A (PKA) mediate a cAMP-induced decrease in intracellular calcium resulting in relaxation and vasodilation^12^. Additionally, PKA mediates the phosphorylation of the nuclear CREB-binding proteins to stimulate the expression of numerous genes to reduce smooth muscle cell proliferation and migration^13^. Both PGI2 and PGE1 are potent pulmonary vasodilators and inhibitors of platelet aggregation. A deficiency in endogenous prostacyclin may be a contributing factor to the pathogenesis of certain forms of PAH^11^. Several studies have suggested that the use of lipid microspheres incorporating PGE1 increases the therapeutic efficacy and prolongs the half-life of PGE1 in the treatment of pulmonary arterial hypertension^14–16^. Additionally, there is evidence that the lungs of PAH patients exhibit decreased expression of the IP receptor^10^. Therefore, the development of stable long-acting prostacyclin analogs and elucidation of the signaling transduction underlying PAH pathology can improve the prospects for long-term pulmonary vasodilator therapy.

cAMP response element binding protein (CREB), a transcription factor, has been identified as a modulator of the vascular smooth muscle cell phenotype and is downregulated in several vascular diseases^17^. CREB reduces mitogen-stimulated vascular smooth muscle cell (VSMC) proliferation, migration, and matrix protein expression and protects smooth muscle cells from apoptosis^17–20^. Decreased levels of CREB protein and the active form of CREB (phosphoserine 133 CREB, pCREB) in medial VSMCs have been observed in rodent models of insulin-resistant and insulin-deficient diabetes-associated vascular disease^18^. Similarly, in a model of pulmonary vascular injury, specifically hypoxia-induced pulmonary hypertension, loss of CREB function is concurrent with pulmonary artery hypertrophy^20^. This can be modeled *in vitro* by exposing PASMCs to PDGF which induces CREB nuclear export and degradation via a pathway downstream of AKT and casein kinase 2 (CK2)^19^.

PTEN is a tumor suppressor gene located on human chromosome 10q23.3 and was originally identified as a candidate tumor suppressor gene based on its high frequency of mutation in a variety of tumors. PTEN can regulate cell growth and apoptosis, interact with the extracellular matrix, and inhibit cell migration, spreading, and focal adhesion^21^. PTEN, a phosphatase, possesses the ability to dephosphorylate proteins and lipids^22–24^. The phosphatase activity function of PTEN is a negative regulator of AKT phosphorylation (pAKT) by the cyclic AMP-dependent protein kinase A (PKA) signaling pathway^24^. Additionally, PTEN can dephosphorylate phosphatidylinositol-3,4,5-triphosphate (PIP3) at the D3 position generating phosphatidylinositol 4,5-biphosphate (PIP2) and leading to a decrease in the cellular levels of PIP3^20^. Because PIP3 is necessary for AKT phosphorylation, active PTEN leads to a decrease in the levels of pAKT, which inhibits AKT-mediated cell proliferation. Furthermore, PTEN protein activity increases p21 levels to downregulate cyclin D1, which coordinates G1 arrest of the cell cycle^25^, inhibits cell division and increases cell apoptosis in addition to inhibiting cell spreading and migration.

Selective chronic deficiency of PTEN in SMCs represents a critical mediator of irreversible pulmonary artery hypertension (PAH)^26,27^. However, the underlying mechanism of CREB and PTEN signaling in PAH has not been clarified. In this study, we investigated the possible involvement of PTEN and its regulatory proteins in PGE1-inhibited PAH. We report here that PGE1 affects the migration and proliferative signaling of PASMCs via the upregulation of pCREB to stimulate PTEN expression. This finding elucidates the underlying mechanism of the PGE1/CREB/PTEN regulatory loop for the prevention of progressive PAH.

## Result

### Expression of CREB and PTEN in human donor and IPAH Lungs

**Figure 1:**
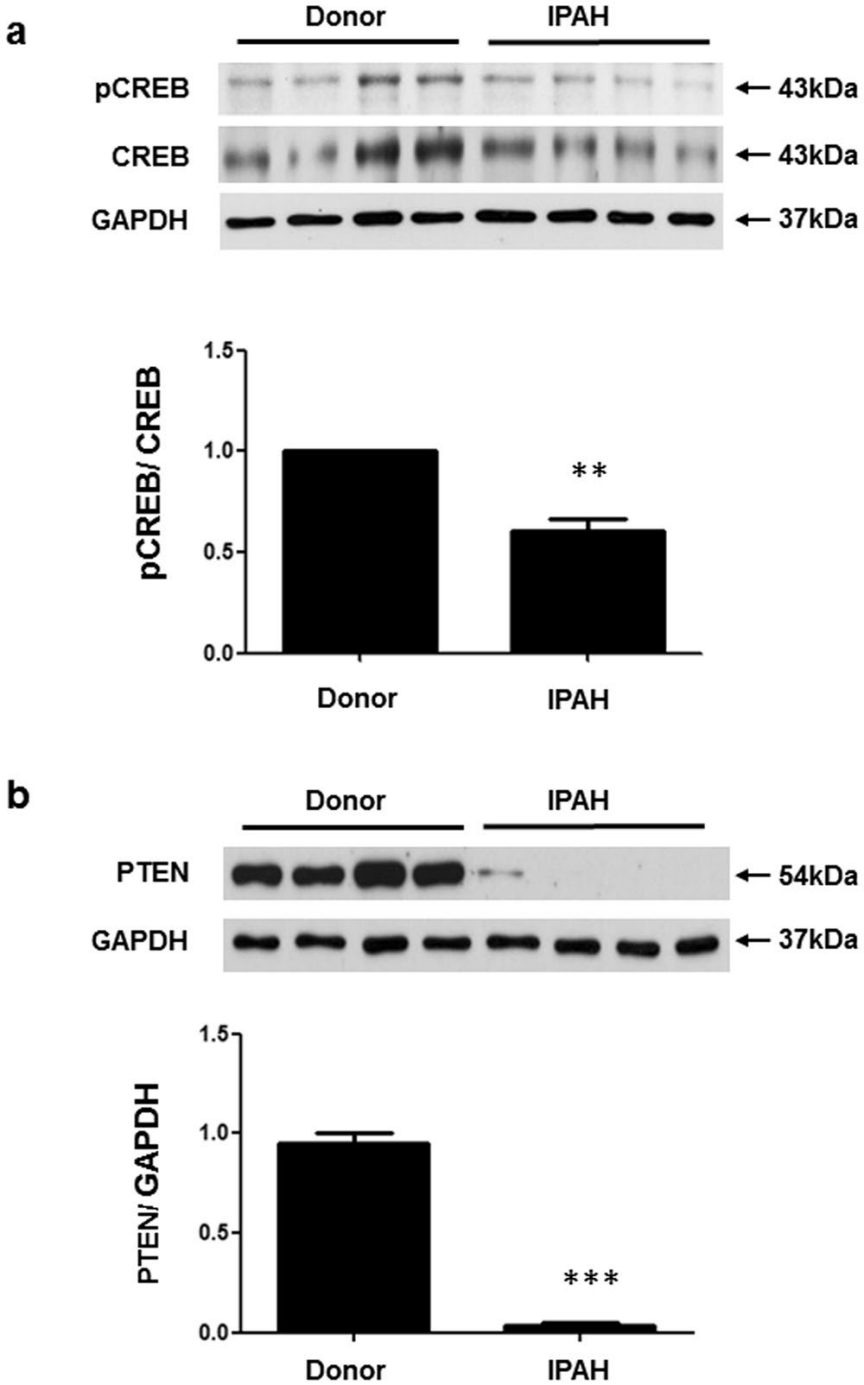
pCREB and PTEN protein levels in human donor and idiopathic pulmonary arterial hypertension (IPAH) lungs. (**a**) pCREB and CREB protein levels were detected in lung tissues as 43-kDa bands and the protein levels decreased in IPAH lung tissues compared with those in donor lung tissues. (**b**) PTEN protein was detected as a 54-kDa band and exhibited scant expression in the IPAH lung tissue compared with that in the donor lung tissue. The bars represent the mean ± SEM of four samples in each group. *P < 0.05 and ***P < 0.001compared with the donor tissue. GAPDH = glyceraldehyde-3-phosphate dehydrogenase.

To determine PTEN and CREB levels in human idiopathic pulmonary arterial hypertension (IPAH) lung tissue, we performed immunoblotting. As shown in the Western blots in Fig. 1a, the pCREB and CREB bands were detected at 43 kDa. The ratio of pCREB to CREB indicated decreased expression in IPAH lungs compared with that in the lungs of human donors. PTEN was detected at 54-kDa and exhibited loss of expression in IPAH lung samples (Fig. 1b). These results reveal that the expression of pCREB and PTEN protein is decreased in IPAH patient tissue compared with that in donor lung tissue.

### Depletion of PTEN activates the AKT signaling pathway in human PASMCs

**Figure 2:**
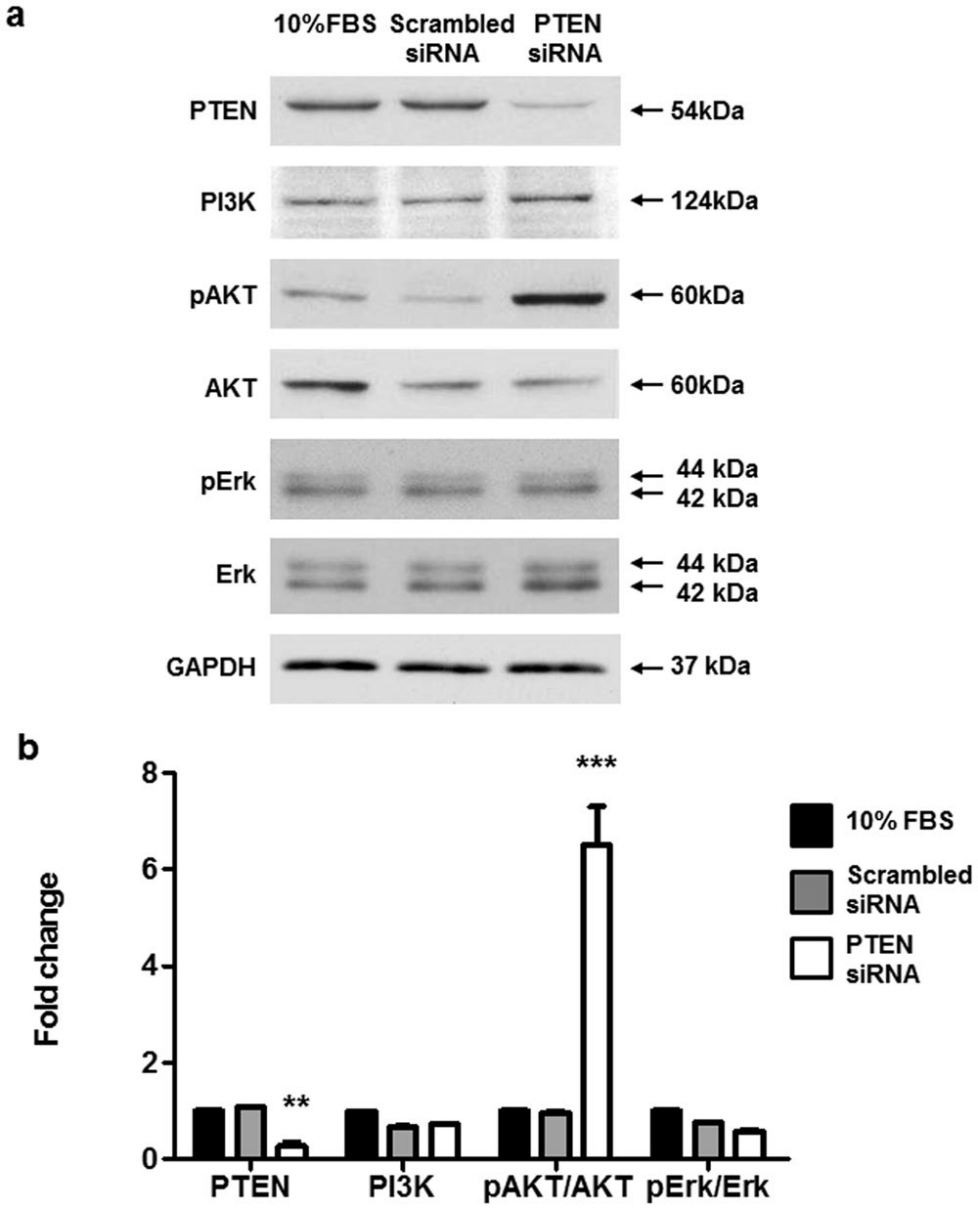
PTEN silencing in control PASMCs induces increased pAKT levels. Commercially available PASMCs were transfected with siRNA for PTEN or control non-targeting siRNA (scrambled siRNA), with 10% FBS used for the control group. Representative immunoblot (**a**) and densitometric quantification (**b**) of protein expression after siRNA transfection. The bars represent the mean ± SEM for n = 3 samples. **P < 0.01, ***P < 0.001 compared with the 10% FBS control group.

The association of defective PTEN with pulmonary arterial remodeling has been investigated in PAH^26^. However, little is known regarding the signaling underlying the changes in relative protein expression in response to defective PTEN in PASMCs. To further assess whether loss of PTEN affects the AKT signaling pathway, we transfected human PASMCs with siRNA to knock down PTEN protein levels and analyzed the transfection efficiency and the relative expression of signaling proteins by immunoblotting (Fig. 2a). The relative protein expression values were obtained by performing densitometry analyses using Image J software (https://imagej.nih.gov/ij/) (Fig. 2b). After PTEN was knocked down, relative expression of the signaling protein PI3K increased slightly, and pAKT increased significantly in PASMCs. It is well known that AKT is a serine/threonine protein kinase and is activated by several growth factors and cytokines in a PI3K-dependent manner^28^. Activation of the PI3K/AKT pathway exerts a major impact on cell proliferation and migration^29,30^.

### PGE1 induces PTEN, pCREB expression and suppresses pAKT

**Figure 3:**
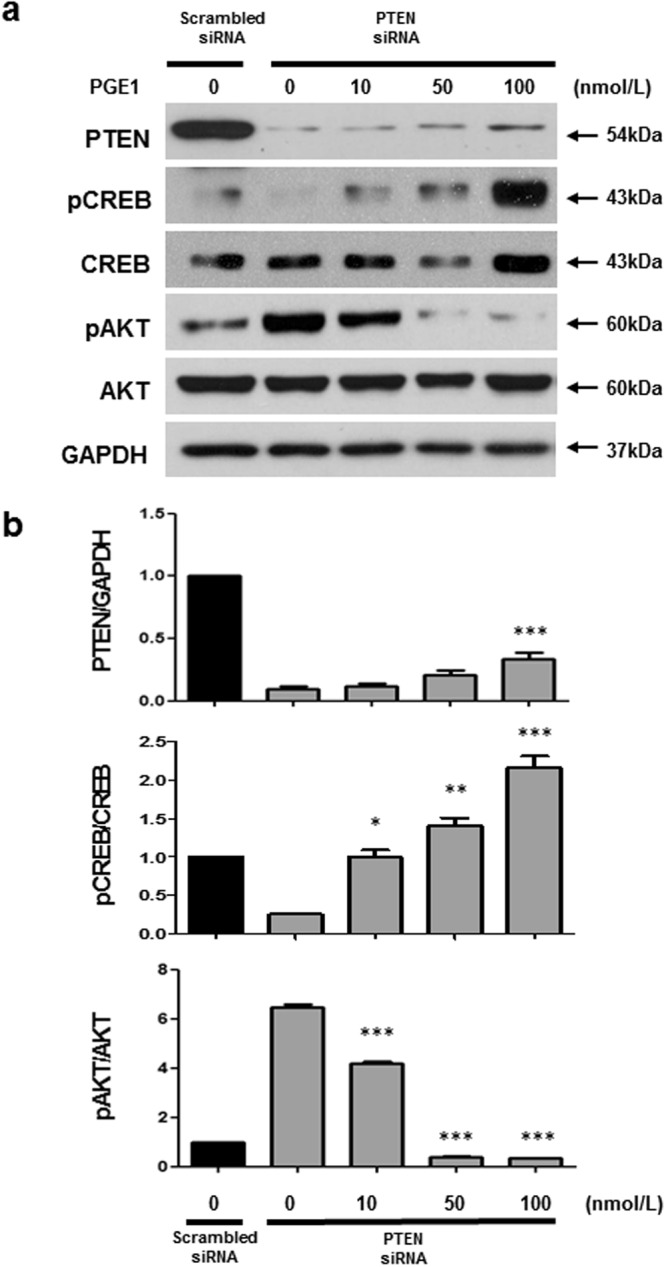
PGE1 induces PTEN upregulation and pAKT downregulation. Commercially available PASMCs were transfected with PTEN siRNA or scrambled siRNA as a control non-targeting siRNA. PASMCs were serum-starved (0.1% serum) for 48 hrs, and then treated with 10, 50, and 100 nmol/L PGE1 for 24 h. Representative immunoblot (**a**) and densitometric quantification (**b**) of protein expression at the indicated dose. The PTEN-silenced PASMCs exhibited increased pCREB and PTEN expression and inhibited pAKT expression in a dose-dependent manner. The bars represent the mean ± SEM. *P < 0.05, **P < 0.01, ***P < 0.001compared with PTEN siRNA only.

**Figure 4:**
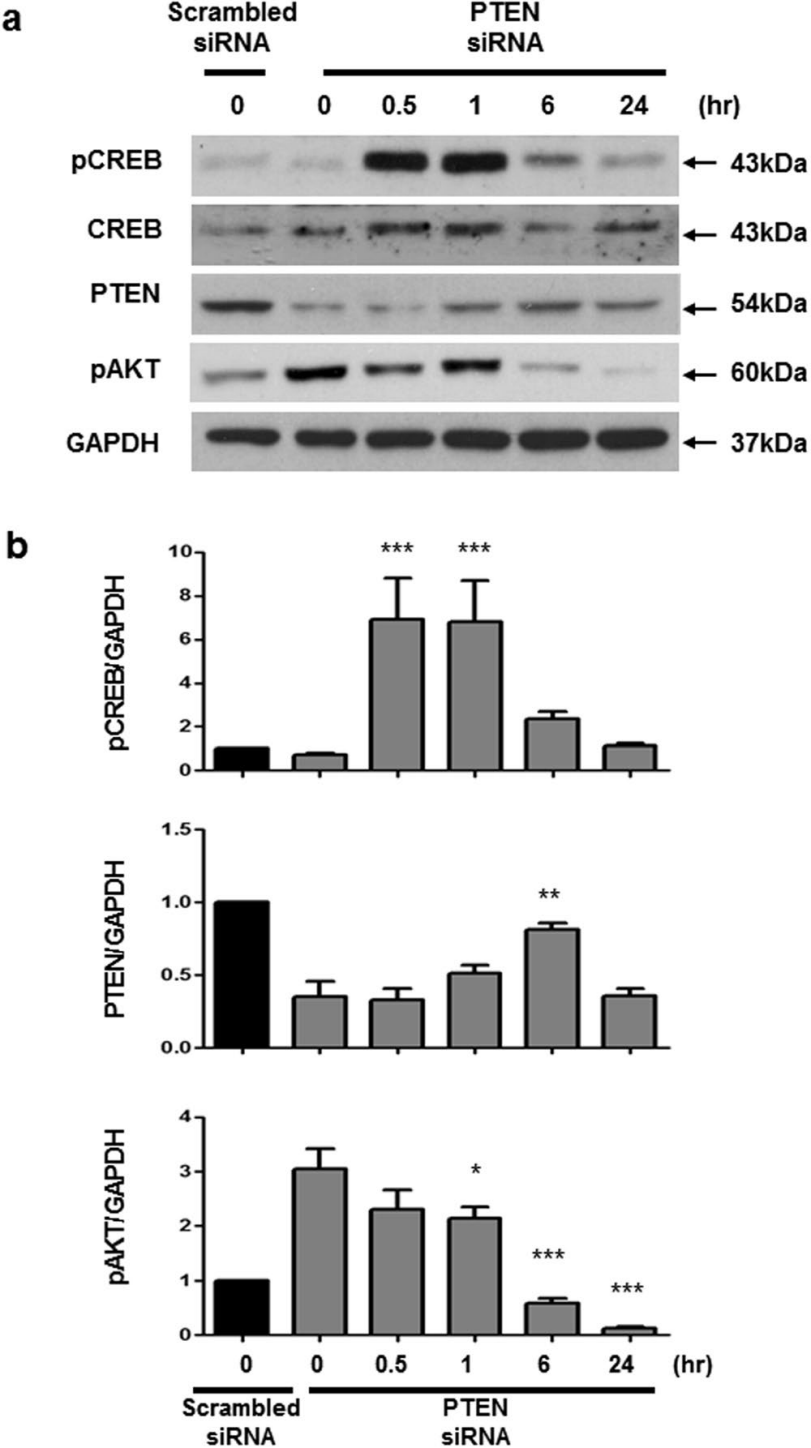
PGE1 induces pCREB and PTEN to suppress pAKT in PTEN-silenced PASMCs. Commercially available PASMCs were transfected with siRNA for PTEN or control non-targeting siRNA (scrambled siRNA). Representative immunoblot (**a**) and densitometric quantification (**b**) of protein expression after siRNA transfection. PGE1 (100 nmol/L) elicited pCREB in PTEN-silenced PASMCs at 30 min, followed by an increase in PTEN expression at 6 h, and a decrease in pAKT from 6 to 24 h. The bars represent the mean ± SEM. *P < 0.05, **P < 0.01 and ***P < 0.001 compared with PTEN siRNA only.

The mechanism by which the depletion of PTEN leads to PI3K/AKT axis deregulation in PAH is unclear, but it has been suggested that PTEN participates in CREB regulation in Hela cells^31^. Additionally, it has been demonstrated that PGE1 acts as a vasodilator for the treatment of PAH^14–16^. The cellular and clinical data implicating potential associations between CREB, PTEN and AKT led us to hypothesize that PGE1 activates PKA-mediated pCREB to induce the inhibition of AKT through PTEN, which is potentially associated with pCREB regulation. To determine whether an association exists between CREB and PTEN, AKT was activated via PGE1 (10, 50, or 100 nmol/L) treatment in PTEN-defective PASMCs. Western blot analysis employing whole cell lysates showed that PGE1 treatment upregulated pCREB and PTEN and downregulated pAKT in a concentration-dependent manner compared with PTEN-defective PASMCs without PGE1 treatment (Fig. 3a, and b). Additionally, in a time course experiment, pCREB levels were initially increased at 0.5 to 6 h and PTEN expression levels were increased from 1 to 6 h. Furthermore, pAKT decreased at 6 h after PGE1 treatment (Fig. 4). Taken together, the results show that PTEN-defective PASMCs increased the pCREB, PTEN, and decreased pAKT expression levels in response to PGE1 treatment in dose and time course-dependent manners. Based on the time course results, we assumed that PGE1 may induce pCREB to activate PTEN, and then inhibit pAKT activation in PTEN-depleted PASMCs.

### The role of CREB in PGE1-induced PTEN expression

**Figure 5:**
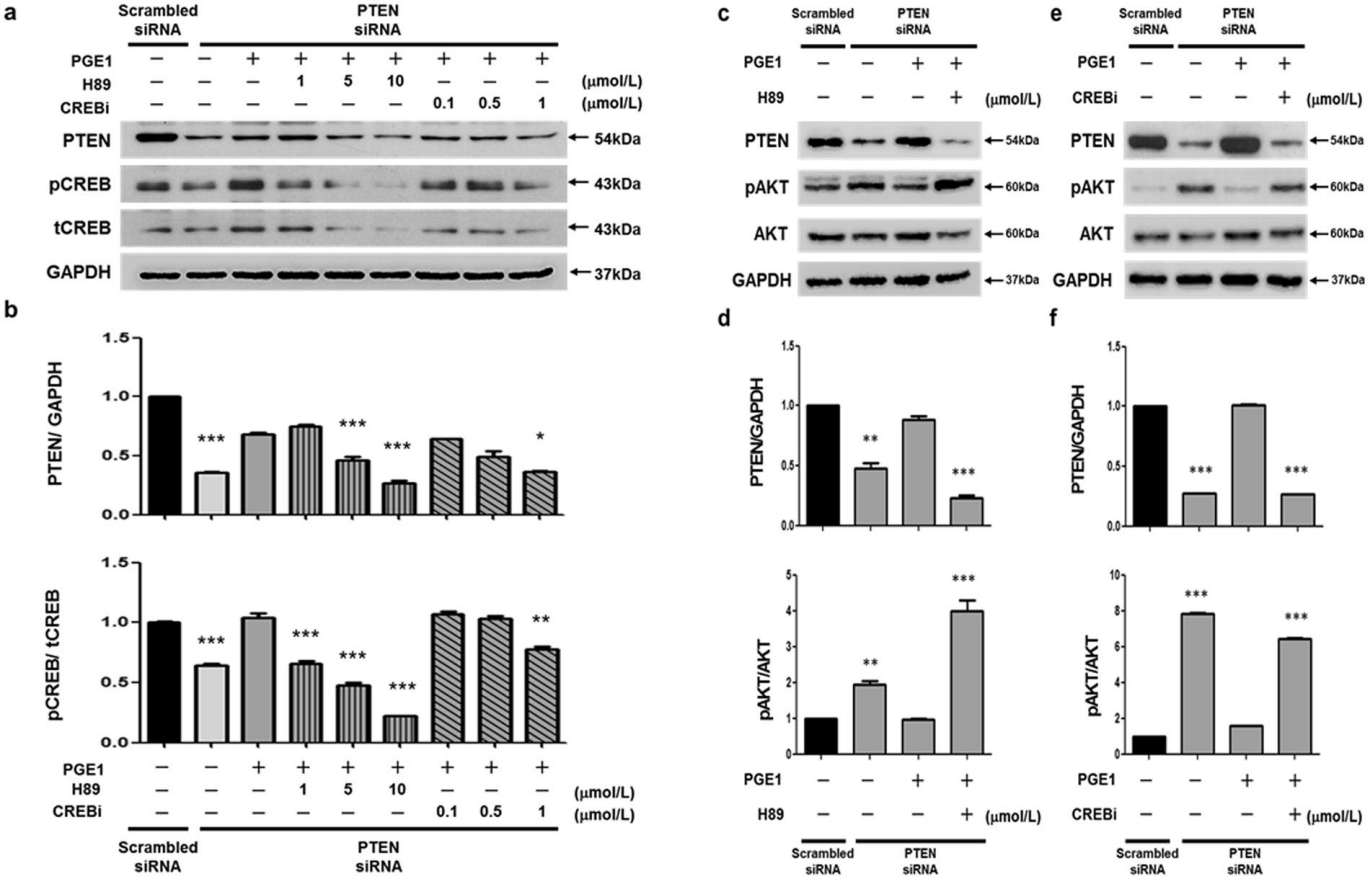
PKA and CREB inhibitors block PTEN expression mediated by PGE1 in PTEN-silenced PASMCs. PASMCs were transfected with siRNA for PTEN or scrambled siRNA as a control non-targeting siRNA. The PTEN-silenced PASMCs were exposed to a PKA inhibitor (1, 5, or 10 μmol/L) or CREBi (0.1, 0.5, or 1 μmol/L) and incubated with or without PGE1 (100 nmol/L). Representative immunoblots of PTEN and pCREB following treatment with 100 nmol/L PGE1 was reversed with a PKA inhibitor (H89) and a CREB inhibitor (CREB i) (**a**, **b**). PTEN-induced inhibition of pAKT via treatment with 100 nmol/L PGE1 was also reversed by the PKA (H89) and CREB inhibitors (CREB i). Representative immunoblots of the H89 (**c**) and CREBi inhibitors (**d**) and densitometric quantification of protein expression following PKA (**e**) and CREBi inhibitor (**f**) treatment in PTEN-silenced PASMCs.

PGE1 is capable of increasing SMC intracellular cAMP levels^12^. Moreover, cAMP induces the PKA pathway to phosphorylate the nuclear CREB-binding proteins^13^. To associate the negative effects of PTEN on the PI3K/AKT signaling pathway with pCREB regulation, we utilized a PKA (H89) and CBP-CREB interaction inhibitors (CREBi) to investigate whether PGE1 attenuates PASMCs by activating the phosphorylation of CREB and the PTEN signaling pathway. CREB, a transcription factor, has been identified as a modulator of VSMC phenotypes and is downregulated in several vascular diseases^17–20^. Decreased levels of CREB protein and the active form of CREB, (phosphoserine 133 CREB, pCREB) in medial VSMCs have been observed in rodent models of vascular insulin-resistant and insulin-deficient diabetes^18^. Activated pCREB recruits its transcription co-activator, CREB-binding protein (CBP), to a cAMP response element (CRE) region in target genes^32^. This recruitment of CBP is a critical step for the transcriptional activation of CREB^33^. Therefore, blocking the interaction between CREB and CBP may be an approach to inhibit CREB activity to assess the role of pCREB in PGE1-induced PTEN expression. Finally, to determine the contribution of CREB to PGE1-induced PTEN expression, we performed additional experiments to assess the role of pCREB in PTEN-defective PASMCs using PKA (H89) and CBP-CREB interaction inhibitors (CREBi) in combination with or without PGE1 treatment. Pre-incubation with H89 blocked the PGE1**-**dependent PKA/pCREB pathway, and CREBi blocked the CBP-CREB function. The PTEN knocked-down PASMCs were exposed to a PKA inhibitor (1, 5, 10 μmol/L) or CREBi (0.1, 0.5, 1 μmol/L) and incubated with or without PGE1 (100 nmol/L) to investigate the expression of pCREB and CREB. The PGE1-induced pCREB and PTEN expression levels were inhibited by H89 (PKA inhibitor) at 1, 5, and 10 µmol/L in a concentration-dependent manner and by the CREB inhibitor at 1 µmol/L compared with PTEN-defective PASMCs without PGE1 treatment (Fig. 5a,b). The PTEN knockdown PASMCs were exposed to CREBi (1 µmol/L) or a PKA inhibitor (10 µmol/L) for 6 h, and then incubated with or without PGE1 (100 nmol/L) for 24 h. PGE1-induced PTEN expression, which inhibited pAKT, was reversed by H89 (PKA inhibitor) (Fig. 5c,d) and the CREB inhibitor (Fig. 5e,f), thus reflecting a crucial role for pCREB and the PKA-dependent pathway in PGE1-induced effects. In contrast, PTEN and pCREB/CREB were stably expressed. Treatment of PASMCs with PGE1 did not significantly affect PTEN and pCREB/CREB expression, but it did affect pAKT. The response to H89 but not CREBi suggests that an alternative PKA-dependent pathway may be important in these cells (see Supplementary Fig. S1). These observations suggest that the PGE1-mediated suppression of pAKT in PTEN-defective PASMCs may be related to pCREB and PTEN through the PKA pathway and CBP-CREB interactions. These results indicated that PGE1 attenuates PASMC by activating the phosphorylation of CREB and the PTEN signaling pathway.

### PGE1 suppresses the proliferation and migration of PTEN-depleted PASMCs by activating phosphorylation of CREB and the PTEN signaling pathway

**Figure 6:**
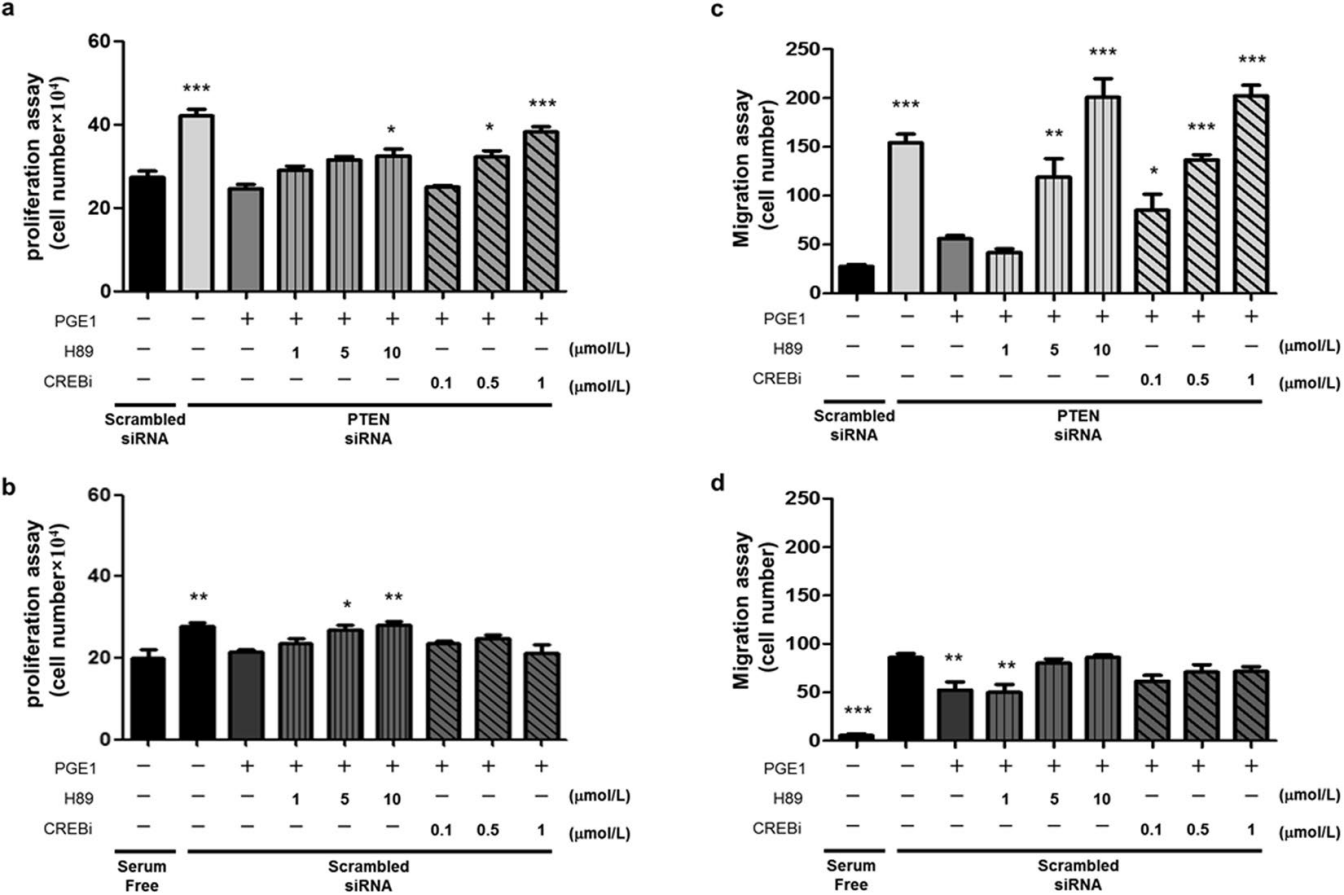
Effect of PGE1 on the silencing of PTEN-induced PASMC proliferation and migration. Commercially available PASMCs were transfected with siRNA for PTEN or control non-targeting siRNA (scrambled siRNA). PTEN-silenced PASMCs were exposed to the PKA inhibitor (1, 5, or 10 μmol/L) or CREBi (0.1, 0.5, or 1 μmol/L) and incubated with or without PGE1 (100 nmol/L). PGE1 inhibited the proliferation and migration of PTEN-silenced PASMCs, which was reversed by a PKA (H89) and CREB inhibitors (CREB i) (**a**, **c**). PGE1 inhibited the proliferation and migration of scrambled-siRNA PASMCs were not dose-dependent reversed by PKA (H89) and CREB inhibitor (CREB i) (**b**, **d**).The bars represent the mean ± SEM, for n = 6 samples. *P < 0.05, **P < 0.01 and ***P < 0.001 compared with scrambled siRNA (**a**, **c**, **d**) or with serum free (**b**).

PASMC proliferation and migration greatly contribute to PAH development^2,3^. Prostaglandin E1 (PGE1) has been demonstrated to be a vasodilator used for the treatment of PAH^14–16^. We next performed an experiment to evaluate whether PGE1 inhibits PAH formation *in vitro*. Furthermore, the PTEN knockdown PASMCs were exposed to a PKA inhibitor (1, 5, 10 μmol/L) or CREBi (0.1, 0.5, 1 μmol/L) and incubated with or without PGE1 (100 nmol/L) to investigate the effect of PGE1 on the proliferation and migration of PTEN-depleted PASMCs. The treatment of PTEN-depleted PASMCs with PGE1 led to a significant decrease in serum-induced PASMC proliferation and migration, but was reversed by H89 and CREBi (Fig. 6a and c). Additionally, treatment with 10 μmol/L H89 or CREBi at 1 μmol/L had non-specific effects on the migration and proliferation of PTEN-depleted PASMCs in the absence of PGE1 (see Supplementary Fig. S2). These data demonstrated that the depletion of PTEN in PASMCs may significantly affect cell proliferation and migration via pCREB and PKA signaling activation. However, when scrambled siRNA-treated PASMCs were exposed to H89 or CREBi and incubated with or without PGE1 (100 nmol/L), the response of the cells was similar to those treated with siPTEN (although the magnitude of the changes was less). While these changes could also be due to increased PTEN expression (an effect more evident in the setting of PTEN knockdown) via PGE1 treatment, the response to H89 but not CREBi suggests that an alternative PKA-dependent pathway may be important in these cells. (Fig. 6b and d). Moreover, treatment with PGE1 can induce PTEN expression to suppress the proliferation and migration of PTEN-depleted PASMCs, but this was reversed by H89 and CREBi, reflecting a crucial role of pCREB and PKA-dependent pathway in PGE1-induced effects. These data support the notion that PGE1 suppresses the proliferation and migration of PTEN-depleted PASMCs by activating phosphorylation of CREB and the PTEN signaling pathway.

### Effects of PGE1 on hemodynamic and structural changes in PAH

**Figure 7:**
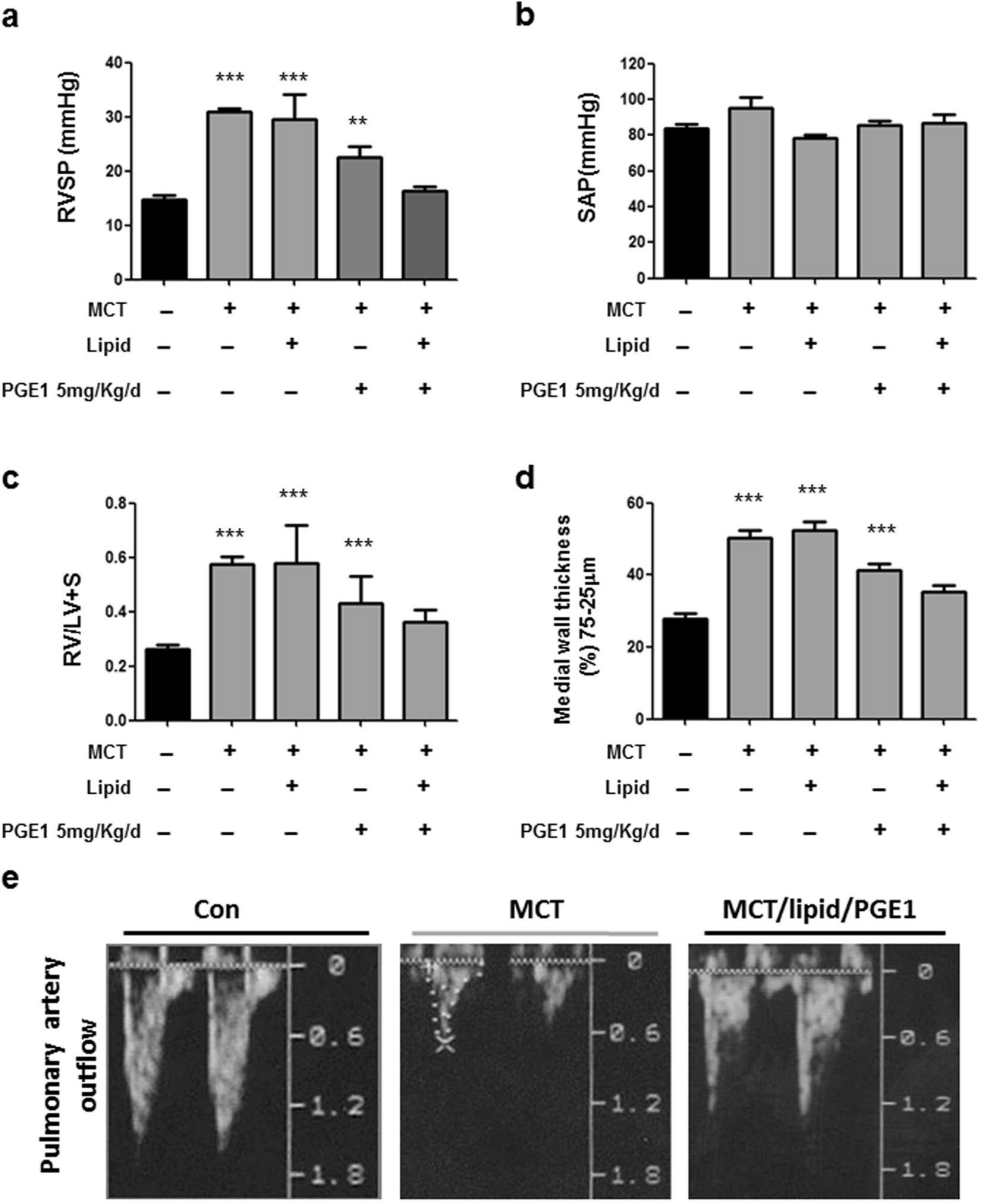
Effects of PGE1 on pulmonary hemodynamics and pulmonary arterial hypertrophy. The right ventricle systolic pressure (RVSP) (**a**) and systemic arterial pressure (SAP) (**b**) measurements for 5 different groups are shown. Pulmonary hypertension (indicated by elevated RVSP) was established 28 days after MCT injection with or without PGE1 treatment and lipid/PGE1 (5 mg/kg/d administered i.p. from day 7 to day 28). The MCT/lipid/PGE1 groups exhibited reduced RVSP compared with the MCT-treated group but not reduced SAP. The ratio of RV to LV plus septum weight (RV/LV + S) is shown (**c**). The medial wall thickness of the small pulmonary arteries (25–75 μm) identified by α-SM-actin staining (brown staining) is shown (**d**). The degree of medial wall thickness was compared among the 5 groups. The mean±SEM (n = 4–8 in each group) is expressed for each group. (**e**) The pulmonary artery outflow measured by echocardiography is shown. ***P < 0.001 versus the control group, one-way ANOVA with Bonferroni’s post -hoc test.

PGE1 has been demonstrated to act as a vasodilator for the treatment of PAH. However, the short half-life of PGE1 in the blood stream and low systemic blood pressure are major issues regarding its use for PAH. In this study, a novel lipid emulsion composition comprising PGE1(lipid/PGE1) (Taiwan Liposome Company, Ltd, Taipei, Taiwan) was employed to prolong the half-life of PGE1 in the blood stream and reduce pulmonary arterial pressure. Sustained release and targeting effects of ePGE1 were expected. The efficacy of ePGE1 (lipid/PGE1) was evaluated in a monocrotaline-induced PAH rat model. Figure 7 displays the results for 26 rats in 5 groups. As expected, rats challenged with MCT indeed developed PAH and right ventricle (RV) hypertrophy, as indicated by increased right ventricle systolic pressure (RVSP) and a right ventricle to left ventricle plus septum (RV/LV + S) weight ratio, respectively, on the 28^th^ day after MCT injection compared with those of controls (Fig. 7a). Administration of lipid/PGE1 from the 8^th^ to the 28^th^ day after MCT injection caused reduced RVSP (Fig. 7a) and decreased the RV/LV + S weight ratio (Fig. 7b); however, no differences in the systemic arterial pressure (SAP) were observed (Fig. 7c). The medial wall thickness (MWT) increased in the small PA wall (75–25 µm) of the MCT group compared with that in the control group (Fig. 7d). In the MCT/PGE1 only and MCT/lipid/PGE1 groups, the lipid/PGE1 treatment resulted in a significant reduction in the MWT of the small PA compared with the MCT group (Fig. 7d), which correlated with the hemodynamic findings. The effect of lipid/PGE1 on PTEN expression in the MCT-induced PAH rat model was evaluated. Consistently, the echocardiography documented the improvement of PA hemodynamics^34^ (including increased PA flow) in the MCT/lipid/PGE1 groups compared with those in the MCT group (Fig. 7e).

### Effects of PGE1 on PTEN expression in PAH

**Figure 8:**
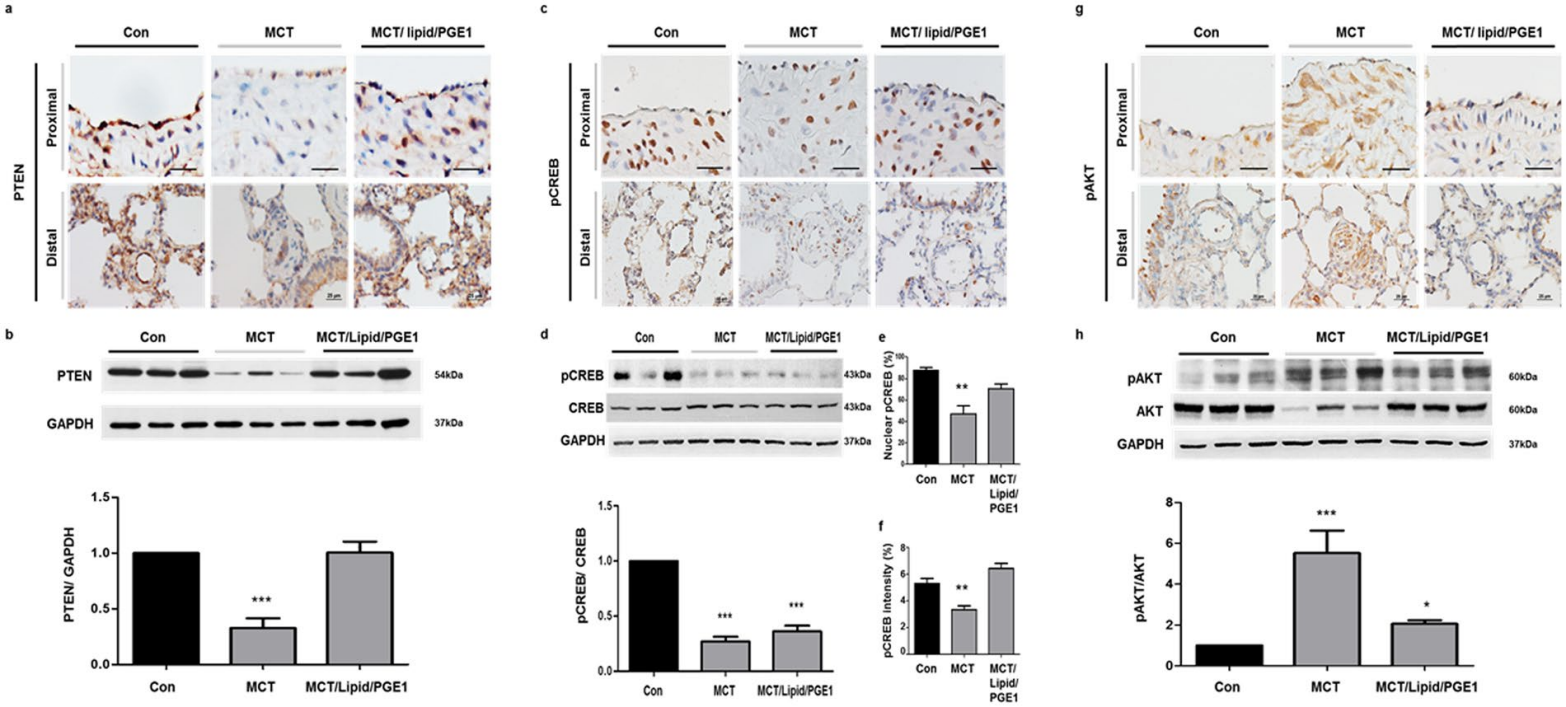
Effects of PGE1 on PTEN expression in the proximal and distal pulmonary arteries of rat lungs. Immunohistochemistry shows (**a**) PTEN, (**c**) pCREB and (**g**) pAKT expression in the proximal (scale bar: 50 μm) and distal (scale bar: 25 μm) pulmonary arteries. PTEN, pCREB and pAKT levels in the medial layer of the pulmonary arteries of the control lungs were identified by brown-colored staining. Representative immunoblotting and densitometric quantification of protein expression in the indicated three groups. (**b**) PTEN was detected as a 54-kDa band; (**d**) pCREB was detected as a 43-kDa band and (**h**) pAKT was detected as a 60-kDa band. (**e**) The nuclear pCREB and (**f**) the intensity of pCREB was detected as a brown staining. The PTEN and pCREB expression levels were decreased but pAKT expression was increased in the pulmonary arteries of the MCT rats. PTEN was elicited and pAKT was diminished in the pulmonary arteries of the MCT rats administered lipid/PGE1. The bars represent the mean ± SEM for n = 3 samples. **P < 0.01 compared with the control (Con) group.

In the MCT-treated rats, medial wall hypertrophy was evident in the muscular pulmonary arteries. The thick medial layer exhibited smooth muscle proliferation. The pulmonary artery from the control rat lung section demonstrated PTEN-positive, pCREB-positive staining, but less pAKT (Fig. 8a,c,g) was observed in the smooth muscle wall of the proximal and distal PA. The MCT rat lung sections exhibited scant PTEN and pCREB staining but exhibited strong pAKT staining (Fig. 8 a,c,g). In contrast, PTEN expression was strongly induced whereas pAKT expression was decreased after treatment with lipid/PGE1 (5 mg/kg/d) (Fig. 8a,c,g), and pCREB overexpression was identified in the nuclei of PASMCs (Fig. 8c,e,f). However, immunoblotting showed that protein levels of pCREB/CREB were increased slightly, but not significantly, increased in the rat lung sections after PGE1 treatment (Fig. 8d), which may be associated with variations in the cellular composition of the lung tissue. Therefore, we utilized Image J to determine the percentage of nuclear pCREB-positive cells among the total number of pulmonary arterial cells (Fig. 8e) and to quantify the intensity of pCREB staining (Fig. 8f) in the pulmonary arterial area. The ratio of nuclear pCREB-positive cells and the pCREB staining intensity were decreased in the MCT rat lung sections compared with controls, but they were increased after treatment with lipid/PGE1 (5 mg/kg/d). The ratio of protein to glyceraldehyde-3-phosphate dehydrogenase (GAPDH) expression indicated decreased expression of PTEN and pCREB but increased expression of pAKT in MCT lungs compared with control lungs (Fig. 8b,d,h). The PTEN and pCREB protein levels, determined via immunoblotting, corroborated the decrease found in idiopathic PAH lung tissue. PTEN expression was strongly elicited and pAKT was decreased following lipid/PGE1 treatment at 5 mg/kg/d; however, pCREB was slightly elicited by PGE1, suggesting that pCREB was specifically increased in the nuclei of PASMCs by PGE1. Taken together, these results demonstrate a reversal in PTEN suppression by PGE1 in the PAH animal model.

## Discussion

This study provides evidence of the promotion of PTEN expression by PGE1 via activation of PKA/CREB cascades to inhibit pAKT level. Additionally, when using PGE1 for therapeutic prevention in the MCT-induced PAH model, significant PTEN induction and pAKT reduction were observed at pulmonary arteries of MCT-induced PAH rats.

Pulmonary artery hypertension is a complex process in which long-term exposure of the vessel wall to vascular insults induces a vicious cycle of pulmonary arterial pathology^2,3^. PTEN has been reported to be important for physiological function and destabilization in tumourigenesis^35^, and loss or inactivation of PTEN has been observed in the setting of PH^31,36,37^. Thus, we chose to focus on the PTEN/AKT pathway. Our study design allowed us to determine whether PTEN is a critical mediator of PAH. To establish a causal role for the scant PTEN in the progression of PH, we deleted PTEN by employing small interfering RNA in human control PASMCs, and documented an increase in the pAKT pathway but not in the ERK signaling pathway. Furthermore, PTEN could inhibit the AKT signaling pathway to attenuate the proliferation and migration of human PASMCs and pulmonary arterial remodeling^26,27,38,39^. We initially hypothesized that PGE1 elicited pCREB and PTEN to inhibit AKT to attenuate the proliferation and migration of PASMCs and pulmonary arterial remodeling in PAH. Indeed, we found that the loss of PTEN in PASMCs was more important for the regulation of the cellular motility than for proliferation. PTEN has been demonstrated to play a conserved role in determining cell polarity in diverse species and cell types, including *dictyostelium discoideum*, neutrophils and neurons^21^; however, there is little information concerning its role in PASMCs. PTEN localizes to the apical plasma membrane during epithelial morphogenesis and catalyzes the conversion of PtdIns (3,4,5)P_3_ to PtdIns (4,5)P_2_. PtdIns (4,5)P_2_ recruits annexin 2 (ANXA2), which in turn attracts CDC42 to the apical plasma membrane for binding to the partitioning defective 6 (PAR6)–atypical PKC (aPKC) complex to promote the establishment of polarity^40^. Thus, the loss of PTEN may prevent normal development of the apical surface and lumen^21,40^.

Downregulation of the transcription factor, CREB, has been reported in several vascular diseases such as atherosclerosis, pulmonary hypertension, insulin resistance and obesity -induced vascular SMC CREB downregulation^19,41^. In pulmonary circulation, the CREB content was found to be high in proliferation-resistant medial subpopulations of smooth muscle cells and low in proliferation-prone regions^20,41^. In chronic hypoxia, the CREB content was depleted and SMCs proliferation was accelerated^20^. Overexpression of wild-type or constitutively active CREB in primary cultures of SMCs arrested cell cycle progression and decreased the expression of multiple cell cycle regulatory genes, as well as genes encoding growth factors, growth factor receptors, and cytokines^20^. PTEN has a well-known role as a negative regulator of the PI3K/AKT pathway and previously been considered to regulate CREB through inhibition of AKT^24,31^. Additionally, PTEN activity can be regulated post-translationally by acetylation and oxidation. PTEN appears to be acetylated at Lys125–Lys128 by p300/CREB-binding protein (CBP)-associated factor (PCAF; also known as KAT2B) and at Lys402 by CBP. Acetylation of PTEN inhibits its catalytic activity and enhances its interaction with PDZ domain-containing proteins^42^. However, the impact of active pCREB on the progression of PAH remains unclear in the context of decreased PTEN and pCREB in lung tissue from patients with PH. PGE1 elicits signaling by binding to select Gs protein coupled surface PGE (EP) 2 and 4 receptors or the prostacyclin (IP) receptor, and receptor activation results in increased intracellular levels of cyclic 3′,5′-adenosine monophosphate (cAMP)^43^. Elevation of cAMP stimulates the expression of numerous genes through the protein kinase A (PKA)-mediated phosphorylation of nuclear cAMP response element binding proteins (CREB)^43^. Furthermore, the PKA inhibitor H-89 blocks the phosphorylation of CREB^44^. To evaluate the contribution of CREB to PGE1-induced PTEN expression, additional functional experiments were performed in human PASMCs via siRNA-mediated depletion of PTEN using a PKA inhibitor (H89) and a CBP-CREB interaction inhibitor (CREBi). PGE1-induced PTEN expression was inhibited by the PKA inhibitor and the CBP-CREB interaction inhibitor, clearly demonstrating the contribution of CREB-PTEN in mediating the effects of PGE1. PGE1 stimulates cAMP-PKA signaling to induce pCREB to upregulate PTEN, leading to AKT signaling inhibition. In contrast, PTEN and pCREB/CREB were stably expression. Treatment of PASMCs with PGE1 did not significantly affect PTEN and pCREB/CREB expression, but it did affect pAKT. The response to H89 but not CREBi suggests that an alternative PKA-dependent pathway may be important in these cells.

Current therapeutic strategies for PAH involve the use of short-acting inhalable or injectable formulations of anti-PAH drugs^1,35,45^. Unfortunately, the pharmacotherapeutic approaches for PAH have many disadvantages, including a requirement of 9–12 inhalations a day (Ventavis^®^, Iloprost inhalation solution) due to short drug half-lives, and response desensitization^45,46^. PGE1 is a potent pulmonary vasodilator with a very short biological half-life of 3–5 min. Recently, several novel drug delivery systems were developed to overcome the limitations of the short duration of action and metabolic instability of an investigational anti-PAH drug, PGE1^15,16^. Treatment of PGE1 inhibited the proliferation and migration of human PTEN-depleted PASMCs. Supplementation with a novel emulsion composition comprising prostaglandin E1 (lipid/PGE1) (a gift from TAIWAN LIPOSOME COMPANY) in rats with monocrotaline-induced PAH prevented pulmonary arterial remodeling and improved hemodynamics by inducing PTEN expression.

We conclude that PGE1 elicits pCREB/PTEN to diminish the migration and proliferation of PAH-derived PASMCs. This finding elucidates the relevant underlying mechanism of the PGE1/CREB/PTEN signaling pathway in the prevention of progressive PAH.

## Methods

### Patient characteristics

Human lung tissues were obtained from four donors and four patients with IPAH undergoing lung surgery at National Taiwan University Hospital and informed consents was obtained from all subjects. Lung tissues were snap-frozen after transplantation for protein extraction. The study protocol for tissue donation was approved by the Human Research Ethics Committee at National Taiwan University Hospital (Institutional Review Board 201409069RINA) and Chang Gung Memorial Hospital (Chang Gung Medical Foundation Institutional Review Board 104-0287B) and conducted in concordance with the principles of the Declaration of Helsinki.

### Human pulmonary arterial smooth muscle cell culture

Human PASMCs were obtained from a commercial sources (Lonza). PASMC culture was performed by employing enzymatic digestion methods. Cells were grown in SMC growth medium (5% FBS, 1 µg/ml hydrocortisone, 10 ng/ml human epidermal growth factor, 3 ng/ml basic fibroblast growth factor, 10 µg/ml heparin, 10 µg/ml gentamycin, and 0.25 µg/ml amphotericin) (Lonza), subcultured at a 1:4 ratio in 100 mm dishes (Corning), and used between passages 4 and 8. The cells were starved in SMC starvation medium (0.1% FBS) for 48 h before the experiments. The PASMC phenotype in the isolated cells was confirmed with positive immunocytochemistry employing antibodies against SM-α-actin (Cell-Signaling).

### Experimental design

Adult male Sprague-Dawley rats (200–250 g body weight) were randomized for treatment 28 days after a single subcutaneous injection of 60 mg/kg monocrotaline (MCT) (Sigma) to induce pulmonary hypertension (experimental groups) or of saline alone (control group). In addition to a group of untreated rats, the experimental groups included rats that received once-daily intraperitoneal injection of lipid/PGE1, PGE1, or lipid only (gifts from YF Lin and P. Ken, Taiwan Liposome Company, Ltd, Taipei, Taiwan), at a dose of 5 mg/kg/day for 3 weeks. The control group received only saline. All rats were cared for in accordance with the Chang Gung University Animal Policy following the Guide for the Care and Use of Laboratory Animals. All animal experiments were reviewed and approved by the Chang Gung University Institutional Animal Care and Use Committee (IACUC) (permit number: CGU11–129).

### Hemodynamic measurements and cardiovascular evaluation

Hemodynamic data were obtained on the 28^th^ day after MCT injection. For hemodynamic monitoring, rats were anesthetized via an intraperitoneal injection of urethane (2.5 mg/kg). The right jugular vein was cannulated, and a 1.6 F catheter-tipped pressure transducer (Scisense, Canada) was inserted through the right jugular vein to measure the right ventricular systolic pressure (RVSP). After the rats were sacrificed, the left lung was fixed for histology in 10% neutral buffered formalin, and the right lung was snap-frozen in liquid nitrogen. To assess right ventricular hypertrophy, the RV was separated from the left ventricular (LV) wall and ventricular septum. The wet weight of the RV and free LV wall with ventricular septum were determined. RV hypertrophy and data were reported as the ratio of the RV wall and LV free wall plus ventricular septum (LV + S).

### Assessment of medial wall thickness (MWT)

Fixation was performed by immersing the lungs in 4% paraformaldehyde solution. After paraffin embedding, 3-μm lung tissue sections were incubated with antibodies against α-smooth muscle actin for 1 h. The streptavidin-biotin system (Dako) was used to detect the signals, and brown color development was evaluated following incubation with diaminobenzidine substrate-chromogen for 1 min. The lung specimens were stained to detect α-smooth muscle-actin and examined to evaluate vascular medial hypertrophy. The MWT percentage was used to represent medial hypertrophy. Under 400X microscopic examination, MWT was defined as the distance between the internal and external elastic laminae as calculated using NIS Elements imaging software from Nikon. For vascular sections, the diameter was defined as (longest diameter + shortest diameter)/2. For each of the groups, 100–120 slides were examined by 400X microscopy examination.

### PTEN small interfering (si) RNA

Chemically synthetic siRNA for human PTEN and its control siRNA were purchased from Dharmacon (Lafayette, CO) and transfected into PASMCs using Lipofectamine® 2000 (Invitrogen) according to the manufacturer’s instructions. The knockdown efficiency was evaluated 48 h later by measuring protein levels in lysates via Western immunoblotting (see Western blot analysis).

### Immunohistochemistry

Before immunostaining, the slides were deparaffinized (xylene), washed with alcohol (100%, 95%, 75%, 50% and 35%) and then rehydrated in deionized water. An antigen retrieval protocol was performed as follows^47^: the slides were incubated at 98 °C for 20 min in target retrieval solution pH 9 (Tris/ethylene diamine tetra-acetate buffer, pH 9, Dako Cytomation) and then cooled to room temperature before incubation with 2% Triton-100 for 10 mins. The slides were incubated for 10 min in −20 °C methanol within 3% hydrogen peroxide. After rinsing in 1X PBS, the sections were blocked with 1% goat serum and 1% BSA, followed by overnight incubation at 4 °C with a polyclonal rabbit anti-PTEN antibody (dilution 1:100, Novus), polyclonal rabbit anti-pCREB antibody (dilution 1:100, Cell Signaling), or polyclonal rabbit anti-pAKT antibody (dilution 1:100, Cell Signaling) in Tris-HCl buffer antibody diluent (Dako). The slides were rinsed with 1X PBS and incubated for 30 min with the Dako labeled streptavidin-biotin system (Dako) to detect the signals; brown color development was evaluated following incubation with diaminobenzidine substrate-chromogen for 10 mins. (EnVision/HRP, Dako). Finally, after rinsing with deionized water, the slides were counterstained with hematoxylin, dehydrated, mounted and cover-slipped.

### Western blot analysis

For Western blotting, immunoblotting was performed with anti-PTEN (Cell Signaling), anti-pCREB, CREB, pAKT, AKT (Santa Cruz Biotech), and PI3K (Cell Signaling) primary antibodies. Secondary antibodies were specific for peroxidase-conjugated anti-mouse IgG or anti-rabbit IgG (Sigma-Aldrich) as needed. The blots were visualized using an enhanced chemiluminescence detection system (Amersham). The samples were normalized to GAPDH (Cell Signaling) and densitometric analysis for protein quantification was evaluated with “Image J” software.

### Cell proliferation assay

Cells were seeded at 2 × 10^5^ cells per well on 6-well plates in DMEM/10%FBS culture medium and allowed to adhere overnight. After the cells reached 70% confluence, PASMCs were transfected with PTEN siRNA or a control scrambled siRNA for 6 h followed by removal of the medium and addition of fresh smooth muscle culture medium overnight. The PTEN knocked-down PASMCs were exposed to a PKA inhibitor (1, 5, or 10 μmol/L) or CREBi (0.1, 0.5 or 1 μmol/L) and incubated with or without PGE1 (100 nmol/L) for 24 h. The PASMC were gently trypsinized, and the cell numbers were evaluated following trypan blue staining.

### Cell migration assay

Transwell filter chamber (Corning Costar) with an 8.0-µm pore size were used for migration assays. PASMCs were seeded at a density of 5 × 10^5^ cells per filter. To initiate chemotaxis, cells in 200 µl of DMEM without FCS were added to the upper chamber, and the lower chamber was filled with 600 µl of DMEM. Then, 10% FCS was utilized as a chemotaxis factor to induce cell migration. The PTEN knocked-down PASMCs were exposed to a PKA inhibitor (1, 5, or 10 μmol/L) or CREBi (0.1, 0.5 or 1 μmol/L) and incubated with or without PGE1 (100 nmol/L). The PASMCs were allowed to migrate for 4 h at 37 °C in an atmosphere containing 95% air/5% CO_2_. Then, the cells below the filter membrane were stained with Liu’s stain^48^. The total filter membrane was divided into 6 fields. Each field was randomly photographed and the cell numbers were counted based on visualization of pink color staining^48^.

### Statistical analysis

The mean and standard error (SE) were used to describe the data. Differences between the two groups were determined by performing an unpaired t-test. For multiple groups, one-way ANOVA with a post-hoc Bonferroni test or Dunnett’s test were used to compare data between the groups. A value of P ≤ 0.05 was considered statistically significant.

## Electronic supplementary material

Supplementary Information
