# Author Correction: Prostaglandin E1 Attenuates Pulmonary Artery Remodeling by Activating Phosphorylation of CREB and the PTEN Signaling Pathway

**DOI:** 10.1038/s41598-018-28951-4

**Published:** 2018-07-24

**Authors:** Ying-Ju Lai, Hsao-Hsun Hsu, Gwo-Jyh Chang, Shu-Hui Lin, Wei-Jan Chen, Chung-Chi Huang, Jong-Hwei S. Pang

**Affiliations:** 1 Department of Respiratory Therapy, Chang Gung University College of Medicine, Tao-Yuan, 33353 Taiwan; 2 Cardiovascular Division, Chang Gung Memorial Hospital, Tao-Yuan, 33353 Taiwan; 3 Division of Thoracic Surgery, Department of Surgery, National Taiwan University Hospital and National Taiwan University College of Medicine, Taipei, 10002 Taiwan; 4 Graduate Institute of Clinical Medical Sciences, Chang Gung University College of Medicine, Tao-Yuan, 33353 Taiwan; 5 Division of Thoracic Medicine, Chang Gung Memorial Hospital, Tao-Yuan, 33353 Taiwan; 6 Respiratory Care, Chang-Gung University of Science and Technology, Chia-Yi, 61363 Taiwan; 7 Department of Physical Medicine and Rehabilitation, Chang Gung Memorial Hospital, Linkou, Taoyuan City, Taiwan

Correction to: *Scientific Reports* 10.1038/s41598-017-09707-y, published online 30 August 2017

The original version of this Article omitted an affiliation for Jong-Hwei S. Pang. The correct affiliations are listed below:

Graduate Institute of Clinical Medical Sciences, Chang Gung University College of Medicine, Tao-Yuan, 33353, Taiwan

Department of Physical Medicine and Rehabilitation, Chang Gung Memorial Hospital, Linkou, Taoyuan City, Taiwan

Additionally, Affiliation 6 for Ying-Ju Lai was omitted in the Supplementary Information of the original version of this Article.

These errors have now been corrected in the HTML and PDF versions of this Article, and in the accompanying Supplementary Information.

