# Supplementary material for: Prostaglandin E1 Attenuates Pulmonary Artery Remodeling by Activating Phosphorylation of CREB and the PTEN Signaling Pathway
Source: Sci Rep. 2017 Aug 30;7:9974. doi: 10.1038/s41598-017-09707-y (PMC5577102; doi:10.1038/s41598-017-09707-y)
Supplement: Supplementary file 1 — Supplementary Information [file 41598_2017_9707_MOESM1_ESM.docx]

**Supplementary Data**

**Prostaglandin E1 Attenuates Pulmonary Artery Remodeling by Activating Phosphorylation of CREB and the PTEN Signaling Pathway**

Ying-Ju Lai^1,2,6^*^,+^, Hsao-Hsun Hsu^3^, Gwo-Jyh Chang^4^, Shu-Hui Lin^1^, Wei-Jan Chen^2^; Chung-Chi Huang ^1,5^; Jong-Hwei S. Pang^4,7+^

*Address for Correspondence:

Ying-Ju Lai

Department of Respiratory Therapy, Chang Gung University College of Medicine,

E-mail: yingjulai@mail.cgu.edu.tw


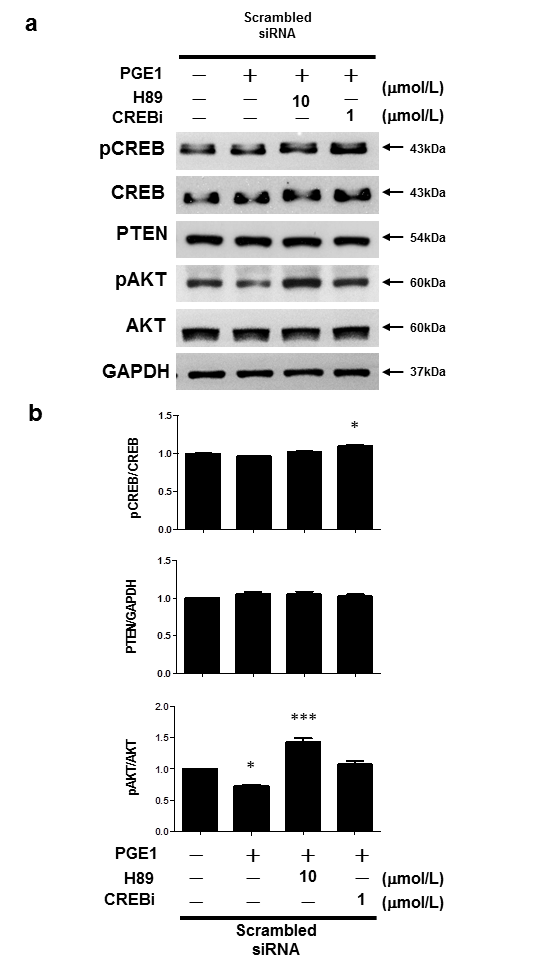


**Supplementary Figure 1. PKA inhibitor (H89) reverses pAKT expression mediated by PGE1 in scrambled siRNA PASMCs.** PASMCs were transfected with scrambled siRNA as a control non-targeting siRNA. The scrambled siRNA PASMCs were exposed to a PKA inhibitor (H89) (10 μmol/L) or CREBi (1 μmol/L) and incubated with or without PGE1 (100 nmol/L). Representative immunoblots of PTEN and pCREB following treatment with 100 nmol/L PGE1 was not changed with a PKA inhibitor (H89) and a CREB inhibitor (CREB i). However, PTEN-induced inhibition of pAKT via treatment with 100 nmol/L PGE1 was reversed by the PKA inhibitor (H89) in scrambled siRNA PASMCs. Representative immunoblots of the H89 and CREBi inhibitors (a) and densitometric quantification of protein expression in scrambled siRNA PASMCs. *P<0.05, ***P<0.001 versus the control group, one-way ANOVA with Bonferroni’s post -hoc test.


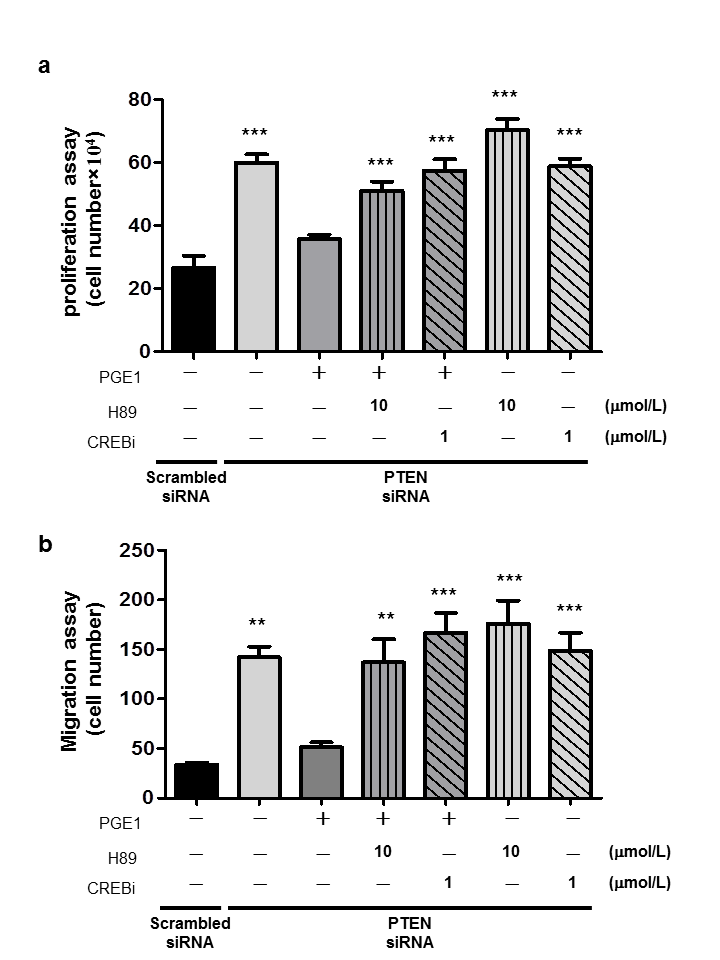


**Supplementary Figure 2. Effect of PGE1 on the silencing of PTEN-induced PASMC proliferation and migration.** Commercially available PASMCs were transfected with siRNA for PTEN or control non-targeting siRNA (scrambled siRNA). PTEN-silenced PASMCs were exposed to the PKA inhibitor (10 μmol/L) or CREBi (1 μmol/L) and incubated with or without PGE1 (100 nmol/L). PGE1 inhibited the proliferation (a) and migration (b) of PTEN-silenced PASMCs, which was reversed by PKA (H89) and CREB inhibitors (CREBi). Additionally, there were non-specific effects of H89 at 10 μmol/L and CREBi at 1 μmol/L on the migration and proliferation of PTEN- silenced PASMCs in the absence of PGE1. The bars represent the mean±SEM, for n=6 samples. **P<0.01, ***P< 0.001 compared with scrambled siRNA group.
